# Acceleration of α-Synuclein Aggregation by Exosomes*[Fn FN2]

**DOI:** 10.1074/jbc.M114.585703

**Published:** 2014-11-25

**Authors:** Marie Grey, Christopher J. Dunning, Ricardo Gaspar, Carl Grey, Patrik Brundin, Emma Sparr, Sara Linse

**Affiliations:** From the Departments of ‡ Physical Chemistry,; ¶ Biochemistry and Structural Biology, and; ‖ Biotechnology,; the § Neuronal Survival Unit, Department of Experimental Medical Science, Wallenberg Neuroscience Center, Lund University, SE-22100 Lund, Sweden and the Center for Neurodegenerative Science,; ** The Van Andel Research Institute, Grand Rapids, Michigan 49503

**Keywords:** {alpha}-Synuclein, Amyloid, Exosome, Fibril, Fluorescence, Lipid, Mass Spectrometry (MS), Membrane, Parkinson Disease, Protein Aggregation

## Abstract

Exosomes are small vesicles released from cells into extracellular space. We have isolated exosomes from neuroblastoma cells and investigated their influence on the aggregation of α-synuclein, a protein associated with Parkinson disease pathology. Using cryo-transmission electron microscopy of exosomes, we found spherical unilamellar vesicles with a significant protein content, and Western blot analysis revealed that they contain, as expected, the proteins Flotillin-1 and Alix. Using thioflavin T fluorescence to monitor aggregation kinetics, we found that exosomes catalyze the process in a similar manner as a low concentration of preformed α-synuclein fibrils. The exosomes reduce the lag time indicating that they provide catalytic environments for nucleation. The catalytic effects of exosomes derived from naive cells and cells that overexpress α-synuclein do not differ. Vesicles prepared from extracted exosome lipids accelerate aggregation, suggesting that the lipids in exosomes are sufficient for the catalytic effect to arise. Using mass spectrometry, we found several phospholipid classes in the exosomes, including phosphatidylcholine, phosphatidylserine, phosphatidylethanolamine, phosphatidylinositol, and the gangliosides GM2 and GM3. Within each class, several species with different acyl chains were identified. We then prepared vesicles from corresponding pure lipids or defined mixtures, most of which were found to retard α-synuclein aggregation. As a striking exception, vesicles containing ganglioside lipids GM1 or GM3 accelerate the process. Understanding how α-synuclein interacts with biological membranes to promote neurological disease might lead to the identification of novel therapeutic targets.

## Teaser

**Background:** Cell-to-cell transmission of α-syn via exosomes has been proposed to propagate Parkinson disease pathology.

**Results:** Exosomes contain gangliosides, several other lipid classes, and proteins. Exosomes and ganglioside vesicles accelerate α-syn aggregation. Vesicles made of other membrane lipids do not.

**Conclusion:** Exosomes provide catalytic environments for nucleation of α-syn aggregation.

**Significance:** Revealing factors that promote α-syn aggregation may provide insight into Parkinson disease pathogenesis.

## Introduction

Extracellular vesicles are released by cells into the extracellular space. In recent years, research into these vesicles has expanded considerably with a major focus on exosomes, defined by their size and the presence of proteins such as Alix and Flotillin-1. These small vesicles of ∼100 nm in diameter are used for intercellular communication. Exosomes are found in most body fluids and contain lipid, protein, and RNA. *In vitro* experiments suggest that exosomal release by neurons depends on synaptic activity and that released exosomes are taken up by other neurons (1).

Several proteins mediate vesicle secretion and trafficking in cells (2). One of these proteins, α-synuclein (α-syn),^5^ appears to play a role in regulating the recycling of synaptic vesicles (3), although its function *in vivo* is poorly understood. The 140-amino acid-long α-syn is expressed at high levels in the brain and accounts for about 1% of the total protein content in the neuronal cytosol (4). α-syn is implicated in Parkinson disease (PD), which is the second most common neurodegenerative disease and leads to an array of motor and non-motor symptoms (5, 6). One neuropathological hallmark of PD is Lewy bodies in which the main component is aggregated α-syn, but other proteins and lipids are also present (7, 8). α-syn is an aggregation-prone protein, and the formation of ordered α-syn aggregates (fibrils) is considered part of the neurodegenerative process in PD and related disorders. The nature of the toxic species is debated; both the smaller aggregates formed during the aggregation process or the process *per se* have been suggested to be toxic (9–13). In solution, α-syn is a largely unstructured protein (14, 15). Membrane-associated α-syn is partly α-helical (15, 16), whereas β-sheet-rich structures form during the aggregation process and are found in Lewy bodies, for example (17, 18).

Compelling genetic evidence supports that α-syn plays a crucial role in PD pathogenesis. Point mutations in the α-*syn* gene cause autosomal dominant PD; α-*syn* gene multiplications are associated with a parkinsonian syndrome, and single nucleotide polymorphisms in the α-*syn* gene are associated with increased risk for PD (19–23).

Emerging evidence suggests that aggregated α-syn spreads from cell-to-cell in a prion-like manner, resulting in transmission of aggregation and neurodegeneration (24, 25). Secreted and lipid-associated α-syn is resistant to proteolysis by the KLK6 peptidase, found in the central nervous system and cerebrospinal fluid, and is co-localized with α-syn in Lewy bodies (26). Secretion of α-syn via exosomes has been proposed to amplify and propagate PD pathology (27–31), and several studies have identified α-syn associated with exosomes (27, 32, 33). Cellular dysfunction is also proposed to increase the transfer of α-syn via exosomes (33, 34). However, the details regarding the interactions between α-syn and exosomes are not understood, and whether exosomes play an important role in PD pathogenesis is still unclear.

In this study, we use a continuous thioflavin T fluorescence assay to investigate the influence of exosomes on α-syn amyloid formation. We find that exosomes accelerate the conversion of monomeric protein to fibrillar aggregates and seek a molecular explanation for this catalysis. In an attempt to explain the acceleration of the process, we determine the molecular composition of isolated exosomes using mass spectrometry, gel electrophoresis, and Western blot, and we investigate the size, charge, and morphology of the exosomes using cryo-electron microscopy, dynamic light scattering (DLS), and ζ potential measurements. We then use this information to design simplified model systems that mimic different aspects of the exosomes, and we explore how they impact α-syn aggregation kinetics. Thus, we examine α-syn aggregation in the presence of exosomes from cells without or with overexpressed α-syn, either wild-type or disease-related mutant protein. We also investigate α-syn aggregation in the presence of artificial vesicles composed of exosome lipid extracts or models composed of biologically relevant lipids, including phosphatidylcholine (PC), anionic phosphatidylserine (PS, common lipid in outer leaflet of the cell plasma membranes), cardiolipin (CL, common in mitochondria), as well as neuro-specific GM1 and GM3 gangliosides and cationic sphingosine. Furthermore, we study the seeding effect by adding small amounts of α-syn fibril seeds. Most experiments are performed at mildly acidic pH to mimic the conditions in endosomes and lysosomes. Under these conditions, the aggregates seem to multiply due to secondary nucleation of monomers on fibrils, a process that is not detected at neutral pH (35).

## EXPERIMENTAL PROCEDURES

### 

#### 

##### Materials

The following lyophilized lipids were obtained from Avanti Polar Lipids (Alabaster AL): 1′,3′-bis(1,2-dioleoyl-*sn*-glycero-3-phospho)-*sn*-glycerol sodium salt (cardiolipin, CL); 1,2-dioleoyl-*sn*-glycero-3-phospho-l-serine sodium salt (DOPS); 1,2-dioleoyl-*sn*-glycero-3-phosphocholine (DOPC); d-erythrosphingosine; and GM1 and GM3 ganglioside from ovine brain and milk, respectively. Buffer chemicals MES, NaCl, and ThT were of analytical grade, and the water used was ultrapure (<18.2 megohms·cm at 25 °C, MilliQ grade).

##### α-Synuclein

Human α-syn was expressed in *Escherichia coli* from the aS-pT7-7 plasmid (kindly provided by Dr. H. Lashuel) and the monomer purified using ion exchange and gel filtration chromatography, as described previously (36), in buffer (10 mm MES, pH 5.5, with 140 mm NaCl, unless otherwise stated) just prior to setting up the kinetic measurements. The protein concentration was determined from the integrated area under the collected peak using the extinction coefficient ϵ_280_ = 5800 liters·mol^−^1·cm^−1^ or by measuring a UV absorbance spectrum from 350 to 220 nm, followed by baseline correction for light scattering effects to obtain the absorbance at 280 nm. The samples were prepared on ice, and 30 μm α-syn (0.4 mg/ml) was supplemented with 20 μm ThT to follow the fibrillation process. α-Syn was also studied in the absence and presence of 0.25 mg/ml exosomes (based on protein content) or 0.1–0.5 mm lipid in the form of small unilamellar vesicles (SUVs). The samples were aliquoted in 96-well plates (nontreated black polystyrene, full volume plates, 3631 Costar) and sealed with a plastic film to avoid evaporation. The pH was measured for all samples and found not to change due to the additions. For seeding experiments, 30 μm α-syn monomer was supplemented with 0.03 or 0.3 μm sonicated fibrils (*i.e.* 0.1 and 1%) counted as monomer equivalents. These fibrils were formed from 30 μm α-syn, collected directly after reaching the final ThT plateau, and sonicated briefly in a sonicator bath to disperse lumped fibrils. Experiments aimed at comparing the seeding efficiency between preformed seeds made in the absence and presence of 0.25 mg/ml exosomes were performed in 96-well plates with a nonbinding surface (black polystyrene plates treated with a pegylated surface, half-area, 3881 Corning Glass). These nonbinding plates were also used to explore the catalytic effect of SUVs composed with ganglioside lipids. Plates were incubated at 37 °C up to 144 h in a FLUOstar Omega or Galaxy plate reader under quiescent conditions or with shaking at 100 rpm between readings.

##### α-Synuclein Solubility

Samples at different time points were aliquoted and centrifuged for 10 min at 13,000 × *g*. The concentration of monomeric α-syn was monitored by UV absorbance at 280 nm for each supernatant, performing at least three measurements per sample, using the Thermo Scientific Nanodrop 2000 spectrophotometer. Specific time points during the lag phase, exponential growth phase, and at the plateau were selected for analysis. These time points were easily pinpointed by following in parallel well controlled and reproducible ThT aggregation kinetics.

##### Cell Culture and Cell Lines

Mouse neuroblastoma cells (N2a) were cultured in Dulbecco's modified Eagle's medium (DMEM) supplemented with 10% fetal calf serum, 100 units/ml penicillin, and 100 μg/ml streptomycin. Cells were incubated in a humidified incubator set at 5% CO_2_ and 37 °C. Plasmids used for α-syn overexpressing lines were described previously (37) with the exception that α-syn was tagged at the C-terminal end with an HA tag. Mutant lines were generated by site-directed mutagenesis. For plasmid transfection, cells were transfected using TurboFect according to the manufacturer's protocol (Thermo Fisher), and stable lines were generated by growing and then maintaining cells in the presence of G418 at concentrations of 200 and 80 μg/ml, respectively.

##### Exosome Purification and Characterization

Exosomes were isolated from cell culture medium as described previously (38). Briefly, N2a cells were cultured for 48 h in serum-free DMEM. Collected medium was then depleted of cells and cellular debris by sequential low speed centrifugation. Exosomes were then isolated by centrifugation of the collected supernatant at 100,000 × *g* at 4 °C for 90 min. The resultant pellet was then washed in PBS and centrifuged for 70 min at 100,000 × *g* at 4 °C. Exosomes were analyzed for size using nanoparticle tracking analysis (NTA) and for purity by Western blotting. The concentration based on protein content was estimated by recording a UV absorbance spectrum from 350 to 220 nm, followed by baseline correction for light scattering effects to obtain the absorbance at 280 nm, and using an approximate absorbance coefficient of 1 liter g^−1^ ^cm−1^. The digestion of exosomes was performed as described previously (32), followed by Western blot analysis.

##### Western Blot

Cell or exosome pellets were lysed directly in Laemmli buffer and boiled for 10 min before samples were applied to 10–20% polyacrylamide gels (Bio-Rad) and subsequent transfer to PVDF membrane using Transblot Turbo transfer packs (Bio-Rad). Membranes were fixed in 0.4% paraformaldehyde (39) before blocking in 5% skim milk powder. The membranes were incubated in primary antibody (HA and Alix (Cell Signaling Technologies), Flotillin-1 (BD Transduction Laboratories), calnexin and GAPDH (Abcam)) solutions overnight. HRP-conjugated secondary antibodies were from Cell Signaling Technologies. Protein bands were detected using Signal Fire ECL reagent (Cell Signaling Technologies) and imaged on Bio-Rad ChemiDoc XRS+.

##### Vesicle Preparation

SUVs were prepared by sonication using a probe sonicator (Vibra-Cell, Sonics). Lipids were dissolved in CHCl_3_/MeOH 2:1 (v/v), and a thin film was formed by air-drying. Any residual solvent was evaporated in a vacuum chamber for at least 12 h. After adding buffer to a final concentration of 1 mm lipid, the samples were vortexed and then sonicated for 10 min using a pulsed sequence (10 s on and 10 s off at 70% amplitude) on ice. Any particulate matter from the probe was removed by 3 min of centrifugation at 13,000 rpm. Exosome lipids were extracted by the Bligh-Dyer method (40), and the SUVs were prepared as above.

##### Light Scattering

DLS and ζ potential measurements were performed using a Malvern Zetasizer Nano ZS (Malvern Instruments, Malvern, UK) at 25 °C.

##### Nanoparticle Tracking Analysis

NTA, monitoring the Brownian motion of vesicles, was performed at room temperature using a NanoSight instrument (Malvern Instruments, Malvern, UK).

##### Cryogenic Transmission Electron Microscopy (cryo-TEM)

To ensure a stable temperature and avoiding loss of solution during sample preparation, a controlled environment vitrification system was used. Samples were prepared as thin liquid films (<300 nm thick) on glow discharge-treated lacey carbon film-coated copper grids and plunged into liquid ethane at −180 °C. In this way, the original microstructures are preserved so that we can avoid component segmentation and rearrangement in addition to water crystallization as the samples are vitrified. Samples were stored under liquid N_2_ until measured and then transferred using an Oxford CT3500 cryoholder and its workstation into the electron microscope (Philips CM120 Biotwin Cryo) equipped with a post-column energy filter (Gatan GIF100). An acceleration voltage of 120 kV was used, and images were recorded digitally with a CCD camera under low electron dose conditions.

##### Lipid Analysis

Extraction of the lipids from the isolated N2a exosomes was carried out by mixing 100 μl of the exosome suspension with 1 ml of CHCl_3_/MeOH (2:1) for 3 h at room temperature. The water phase was removed, and the organic phase was dried in a desiccator. Before being subjected to MS analysis, the dried samples were dissolved in 100 μl of CHCl_3_/MeOH (1:2) and mixed (1:1) with MeOH containing either 10 mm ammonium acetate or 0.4 mm methylamine, reaching a final concentration of either 5 mm ammonium acetate or 0.2 mm methylamine in CHCl_3_/MeOH (1:5).

Samples were analyzed using a nano-electrospray ionizer connected to an Orbitrap-Velos Pro mass spectrometer (Thermo Scientific, Waltham, MA). Samples (∼2 μl) were loaded in disposable emitters and sprayed using negative ionization. Data were collected using data-dependent acquisition performing full scan experiments as well as untargeted MS/MS using higher energy collisional dissociation fragmentation. Relative intensities were calculated within each lipid class by averaging full scan spectra and for each lipid species using the isotope compositions yielding the three lowest masses. The intensities of closely adjacent lipids were compensated according to the theoretical isotopic distributions.

## RESULTS

### 

#### 

##### Exosome Characterization

**FIGURE 1.**
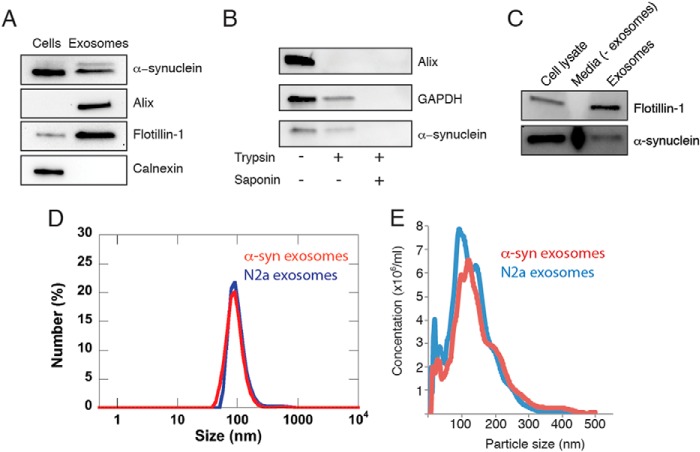
**Characterization of exosomes.** *A,* whole cell lysates and exosomes were subjected to Western blot analysis with antibodies against the proteins indicated. *B,* exosomes were treated with 0.25% trypsin ± 0.1% saponin before Western blotting with antibodies against α-syn, GAPDH (cytosolic protein), and Alix (membrane protein). *C,* relative levels of α-syn in cell lysates compared with that secreted, either free or in exosomes, was analyzed by Western blotting. Cell lysate represents 1% of total cells; media (−*exosomes*) represents 10% of culture medium TCA-precipitated after exosome isolation, and 50% of total exosomes isolated were loaded. *D,* dynamic light scattering of exosomes from N2a cells with or without overexpressing-type α-syn shows vesicles of around 100 nm in diameter (analyzed by number). *E,* isolated exosomes from N2a cells (*blue*) or N2a cells overexpressing wild-type α-syn (*red*) were analyzed by NanoSight nanotracking analysis.

Western blot analysis of the exosome preparations revealed that they were enriched for the exosomal proteins Flotillin-1 and Alix. Calnexin staining of the blots shows that the preparation is free of cellular contamination (Fig. 1*A*). Unlike Flotillin-1 and Alix, the level of α-syn in exosomes is not enriched, suggesting it is not a major protein component of these vesicles. To further confirm the presence of α-syn in exosomes, we digested the exosome sample with 0.25% (w/v) trypsin in the presence and absence of 0.1% (w/v) saponin (Fig. 1*B*). Significant proteolysis was observed when saponin was used to lyse the exosomes; however, the majority of α-syn was found to be protected from digestion in the absence of saponin suggesting α-syn is inside the exosomes. This is consistent with a predominantly cytosolic localization of α-syn in the cells from which the exosomes originate. It has also been reported that only a fraction of α-syn is secreted in association with exosomes (32, 41). To confirm this, cell culture medium was subjected to trichloroacetic acid precipitation after exosome isolation and subsequent Western blot analysis. In comparing the total amount of α-syn found associated with exosomes to that which remains in the culture media after exosome removal (Fig. 1*C*), we also find that only a minor component of total extracellular α-syn associates with exosomes.

The size distribution in the exosome samples was determined using DLS and NTA. The main species in the DLS measurements, representing around 99% of the vesicles by number, was found to have a diameter around 100 nm, which is typical for exosomes (42), and the minor portion (<1%) has a considerably larger diameter (Fig. 1*D*). NTA showed similar results (Fig. 1*E*). The measured ζ potential for exosomes was similar to that obtained for small unilamellar vesicles (SUVs) with anionic lipids (Table 1). We can thus conclude that the exosomes are negatively charged. The cryo-TEM images of the exosomes show unilamellar, spherical vesicles with a diameter of 96 ± 8 (±S.E.), in agreement with the DLS and NTA measurements. Also, a fraction of larger exosomes was visible, with a diameter of 185 ± 35 nm (±S.E.). The membrane thickness was found to be 6 nm, which is typical for cell membranes (normally 4–10 nm) (43, 44). Exosomes from cells overexpressing human α-syn contain dark gray fields (Fig. 10*A*), which may represent protein or some other components of the exosomes. Additional exposure leads to blistering of these fields, further supporting a protein component. However, from these images alone, it is not possible to tell whether this is α-syn, another protein, or several proteins. The cryo-TEM images also show that the vesicles deform when they approach each other, consistent with electrostatic repulsion between the charged membranes.

**TABLE 1:** **ζ potential of α-syn and SUVs used in the kinetic assays and cryo-TEM**

Sample	ζ potential
	*mV*
α-syn exosomes	−16
6% CL SUVs	−21
30% DOPS SUVs	−27
100% DOPC SUVs	−0.5
15% sphingosine SUVs	21
10% GM1 SUVs	−11
α-syn monomers	−1.5
α-syn fibrils	−23

Although we cannot rule out the influence of other extracellular vesicles, our analysis shows that the vesicles isolated meet the criteria for exosomes, and for this reason we will refer to the vesicles we isolated as exosomes. Unless otherwise stated, the term exosomes refers to exosomes isolated from N2a cells overexpressing wild-type α-syn.

##### Reproducible Kinetics Require a Defined Starting State

Central to all the kinetic studies in this investigation is our previously published study where we identified mild conditions resulting in reproducible aggregation kinetics under quiescent conditions or with gentle shaking at slightly acidic pH (36). A key procedure to achieve reproducibility is the gel filtration step used to isolate monomeric protein in degassed buffer. This setup was used previously to explore α-syn interactions with lipids (36, 45) and in this study to characterize the effect of exosomes on α-syn aggregation rate. α-syn has been suggested to exist in a tetrameric state *in vivo*, although the relevance and existence of this form is debated (46, 47). Therefore, isolation of the monomer to obtain a well defined starting state may still be preferred for *in vitro* studies.

The aggregation experiments were performed in slightly acidic conditions. At neutral pH, the α-syn aggregation process is very slow, although it can be accelerated by the vigorous shaking or addition of beads or surfactants (48). When pH is reduced to pH 5.5, the aggregation occurs much faster and can also be reached without agitation (36). Indeed, the rate of secondary nucleation is dramatically increased at mildly acidic pH compared with neutral pH, which explains the acceleration of the aggregation process at the lower pH value (35). This difference in aggregation kinetics can be explained by the reduced electrostatic repulsion between protein units upon the change in pH. At pH 7, α-syn is negatively charged, and when pH is reduced to 5.5, the protein net charge is close to zero (predicted pI 4.74 based on amino acid sequence).

**FIGURE 2.**
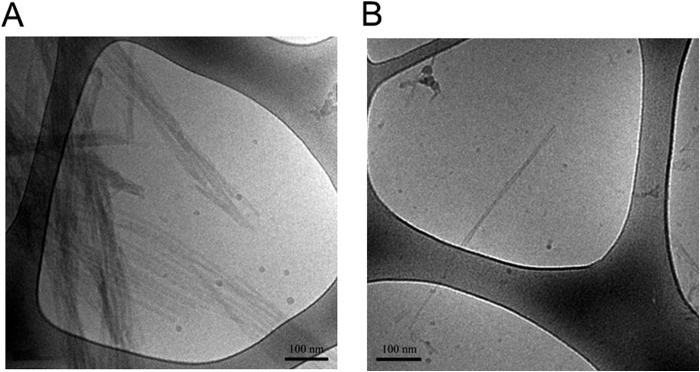
**Formation of α-syn fibrils.** 5 μl of α-syn fibrils in 10 mm MES, pH 5.5, with 140 mm NaCl, unless otherwise stated) was added to the glow-discharged grid for cryo-TEM imaging. *A,* examples of typical α-syn fibrils formed at pH 5.5. *B,* example of a typical α-syn fibril formed at pH 7.5.

ζ potential measurements for monomeric α-syn in 10 mm MES buffer, 140 mm NaCl, pH 5.5, show very low electrophoretic mobility (Table 1), which imply an almost uncharged protein under these solution conditions. Corresponding measurements of fibrillar α-syn were also performed showing higher electrophoretic mobility in the same range as the SUV systems investigated (Table 1). Finally, the fibrils formed at different pH values were investigated by means of cryo-TEM. Fibrils formed from α-syn alone at pH 5.5 (Fig. 2*A*) and pH 7.5 (Fig. 2*B*) appear to be morphologically different, in that the fibrils formed at pH 5.5 are more abundant and bundled, and at pH 7.5 a more distinct inter-filament separation in each fibril is observed.

##### α-Synuclein Aggregation Kinetics in the Presence of Exosomes

α-syn amyloid formation was studied in the absence and presence of exosomes isolated from naive N2a cells or N2a cells overexpressing α-syn. Primary nucleation in solution is extremely slow for α-syn. Experiments in nonbinding plates can therefore only detect acceleration by vesicles. In plain polystyrene plates, the surface promotes nucleation (49). In these plates, we can also observe and discuss catalytic effects, although an observed retardation could arise from a direct effect on the protein or interference with surface nucleation. The aggregation kinetics were followed by ThT fluorescence. When the ThT dye binds to amyloid fibrils and oligomers, it enters a less polar environment, and its rotation is restricted. This gives rise to an increase in fluorescence emission at 482 nm when ThT is excited at 442 nm, but the quantum yield for bound ThT is very sensitive to the detailed fibril structure. The method does not provide direct molecular information, and whereas binding to fibrils dominate the observed signal, the specificity of ThT binding in the pathway of amyloid formation is still not completely understood (50). However, if care is taken in terms of optimized ThT concentration, and a starting point corresponding to monomeric samples in degassed buffers, it is possible to obtain highly reproducible data for the aggregation kinetics (36, 51, 52).

**FIGURE 3.**
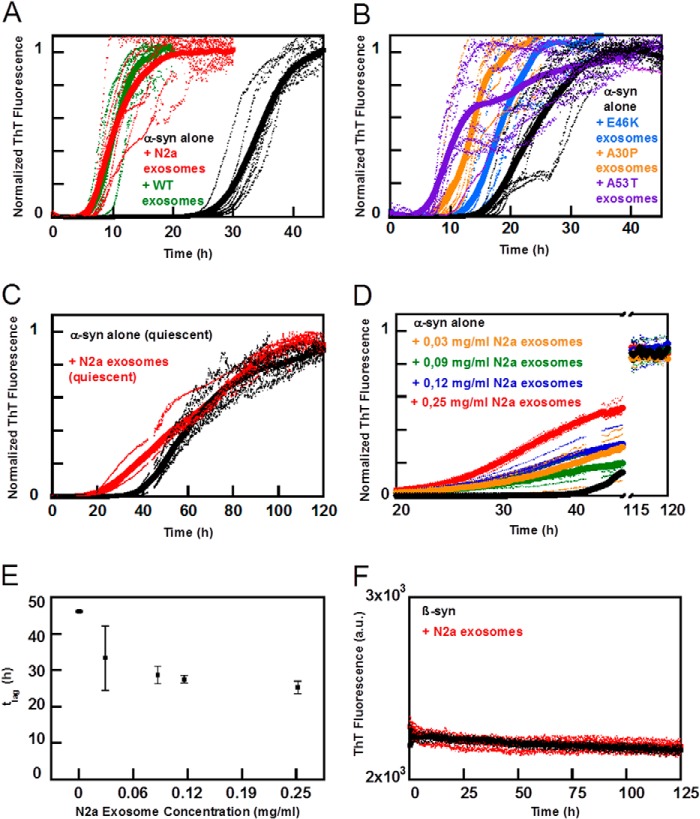
**Aggregation kinetics of α-syn in the presence of exosomes.** Aggregation kinetics for 30 μm α-syn and β-syn was followed by ThT fluorescence in the presence and absence of exosomes in 10 mm MES, pH 5.5, with 140 mm NaCl. The averages of 4–8 replicate traces are shown in *boldface* with individual traces *dashed below. A,* aggregation of α-syn (*black*) with 0.25 mg/ml exosomes from N2a cells (*red*) or N2a cells overexpressing α-syn (*green*) show a distinct difference in lag time. Data were collected at 100 rpm. *B,* aggregation of α-syn (*black*) with 0.25 mg/ml exosomes from cells overexpressing disease-associated mutant α-syn (*purple* A53T, *orange* A30P, and *light blue* E46K) also exhibit a significantly shorter lag time than α-syn alone. Data were collected at 100 rpm. *C,* aggregation of α-syn (*black*) with 0.25 mg/ml exosomes from N2a cells (*red*) under quiescent conditions. *D,* dose dependence aggregation assay of α-syn in the presence of different exosome concentrations varying from 0–0.25 mg/ml. *E,* lag times, corresponding to 10% of maximum intensity, taken from the conditions depicted in *D* pointing again toward a significantly accelerated fibrillation when in the presence of exosomes. *F,* control experiment with β-syn, nonaggregating α-syn homologue protein, which remained unaffected in the presence of 0.25 mg/ml exosomes during the time frame assayed.

Examples of data on the kinetics of amyloid formation starting from 30 μm monomeric α-syn in the absence and presence of exosomes (0.25 mg/ml based on protein content) in 10 mm MES, 140 mm NaCl, pH 5.5, are shown in Fig. 3. We find that under these conditions, exosomes accelerate α-syn aggregation. There is no significant difference between exosomes isolated from naive N2a cells or those overexpressing α-syn (Fig. 3*A*). Furthermore, exosomes from cells that overexpress disease-related mutants of α-syn (A53T, A30P, and E46K) are found to accelerate the α-syn aggregation to the same extent (Fig. 3*B*). From these experiments, we conclude that the half-time for the α-syn aggregation, *t*_½_, in the presence of exosomes is less than one-third of *t*_½_ for α-syn alone. Here, *t*_½_ is defined as the time point for half the maximal elevation of ThT fluorescence relative to baseline. We also observe that the presence of exosomes accelerates the aggregation of α-syn under quiescent conditions, albeit both the perturbed and unperturbed process is slower (Fig. 3*C*). The faster aggregation we observe under shaking conditions is attributed to fibril breakage.

To further elucidate the catalytic effect of exosomes, a dose dependence kinetic assay was performed using exosomes ranging in concentration from 0.03 to 0.25 mg/ml (Fig. 3*D*). At the lowest concentration, we observe a significant decrease in *t*_½_. As increasing amounts of exosomes are added, the lag time is further reduced, but to only a minor extent (Fig. 3*E*), suggesting that the presence of very low amounts of exosomes is sufficient to catalyze α-syn fibril formation. We also investigated whether exosomes would lead to aggregation of β-synuclein (β-syn), a less aggregation prone homologue of α-syn. β-syn was incubated in the presence or absence of 0.25 mg/ml of exosomes. No aggregation was observed during the time frame of the experiment (Fig. 3*F*).

**FIGURE 4.**
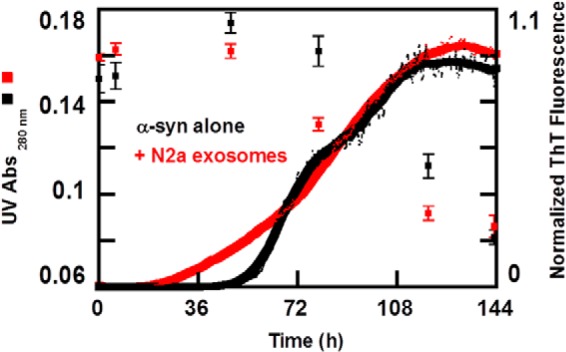
**α-syn monomer concentration during the aggregation process.** Normalized aggregation kinetics for 30 μm α-syn (*black line*) in the presence of 0.25 mg/ml exosomes (*red line*) followed by ThT fluorescence in 10 mm MES, pH 5.5, with 140 mm NaCl. The average traces are shown in *bold*. In parallel to the ongoing aggregation process, solubility changes of α-syn alone (*black squares*) and in the presence of 0.25 mg/ml exosomes (*red squares*) were monitored. Samples were collected at different stages of the aggregation profile and centrifuged, and absorbance measurements were preformed to the supernatant. Each time point therefore represents an average value of at least three repeated absorbance measurements of soluble monomeric α-syn with respective standard deviation bar represented. Well depicted is the correlation between monomer depletion and fibril elongation in the presence and absence of exosomes.

To confirm and complement the aggregation kinetics monitored by ThT fluorescence, we measured the α-syn monomer concentration during the aggregation process. Here, samples were collected at different time points from ongoing aggregation reactions in the absence and presence of exosomes and spun down, and the absorbance of the supernatant was measured at 280 nm. There is a close correspondence between monomer depletion and fibril formation, both in the absence and presence of exosomes (Fig. 4) implying that the dominating species in solution are monomer and fibrils at all times. Moreover, monomer is depleted earlier in the presence of exosomes, confirming the earlier formation of fibrils as deduced from the ThT measurements.

##### Exosome Lipid Composition

The MS analysis revealed the presence of several phospholipid classes in the exosomes, including PC, PS, PE, PI, and ganglioside classes GM2 and GM3, as summarized in Table 2 and supplemental Table SI1. Within each class, we found several species with different fatty acids. The phospholipids were identified by several means. The class and fatty acid composition was determined by MS/MS, through characteristic fragmentation of the headgroup and fatty acids anions. The experimentally observed mass was found to lie within 5 ppm from the theoretical one. The PC lipids were detected as acetate adducts in samples using ammonium acetate, whereas most other lipids could be observed in either ammonium acetate or methylamine. The most abundant PS, PC, PI, and PE species contained 18:1, 18:0, 16:1, and 16:0 acyl chains. Other species were also detected having 20:0–4, 22:0–6, and 24:0–4 in combinations with 18:0–1 or 16:0–1 acyl chains.

**TABLE 2:** **Phospholipid found in exosomes** Phospholipid classes detected are PE, PS, PI, PC, and the gangliosides GM2 and GM3. Fatty acid composition was determined from MS^2^ in which relative intensity is calculated within each lipid class, and where 100% represents the most abundant species found; ppm denotes the deviation of the experimental value from the theoretical.

Class	Fatty acid	Lipid *m*/*z*/ppm	Relative intensity
			%
PE	18:1–16:0	716.5252/2.3	35
PE	18:1–18:1	742.5403/1.4	100
PE	18:1–18:0	744.556/1.5	42
PE	18:1–20:4	764.525/1.9	31
PE	18:0–20:4	766.5408/2.1	71
PE	18:0–20:3	768.5562/1.7	51
PS	16:0–18:1	760.5142/1.0	32
PS	18:1–18:1	786.53/1.2	24
PS	18:1–18:0	788.5454/0.9	100
PS	18:0–20:4	810.5278/−1.5	21
PS	18:1–22:0	844.6096/2.7	67
PI	18:1–18:1	861.5509/1.2	71
PI	18:0:18:1	863.5665/1.2	100
PI	18:0–20:4	885.5516/2.0	36
PI	18:0–20:3	887.5667/1.4	93
PI	18:0–20:2	889.582/1.0	57
PC	16:0–16:2	788.5456/1.1	50
PC	16:0–16:1	790.5606/0.3	21
PC	16:0–16:0	792.5767/0.9	20
PC	16:0–18:1	818.5914/−0.3	100
PC	18:1–18:0	846.6242/1.5	50
GM3	16:0	1151.7080/1.9	52
GM3	20:0	1207.7705/1.7	7
GM3	22:0	1235.8017/1.5	9
GM3	24:1	1261.8172/1.4	12
GM3	24:0	1263.8325/1.1	16
GM2	16:0	1354.7880/2.0	100
GM2	18:0	1382.8184/1.3	8
GM2	20:0	1410.8500/1.5	12
GM2	22:0	1438.8826/2.4	21
GM2	24:1	1464.8972/2.1	31
GM2	24:0	1466.9118/0.9	34

**FIGURE 5.**
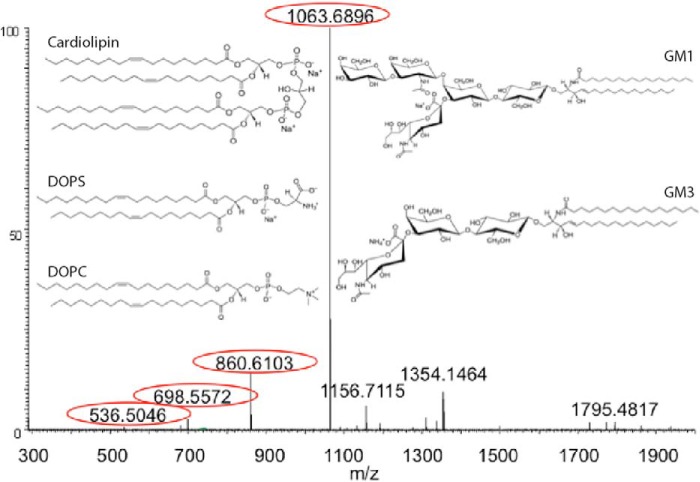
**Lipid identification by mass spectrometry.** Fragmentation of GM species *m*/*z* 1354.8 with a neutral loss of Neu5Ac (−291.1), Neu5Ac + GlcNAc (−494.2), Neu5Ac + GlcNAc + Hex (−656.2), and Neu5Ac + GlcNAc + 2Hex (−818.3) marked in *circles. Insets* show lipid structures as indicated.

Several species of ganglioside were found to have a characteristic fragment of sialic acid (Neu5Ac) with *m*/*z* 290.088. To investigate further, an additional experiment selecting such a lipid *m*/*z* 1354.8 for MS/MS using collision-induced dissociation fragmentation was performed. The mass spectrum of the fragments shows a consequential loss of Neu5Ac followed by *N*-acetylglucosamine (GlcNAc) and two hexoses (Hex), which indicate the identity of a GM2 species with a 16:0 acyl chain (Fig. 5). Similar experiments were also found the presence of several GM3 species. In Table 2, all the GM species found are shown with their relative abundance and class. The most abundant GM2 and GM3 species mostly contained saturated fatty acids, in which 16:0 was dominant (Table 2).

##### α-Synuclein Aggregation Kinetics in the Presence of Model Membranes

**FIGURE 6.**
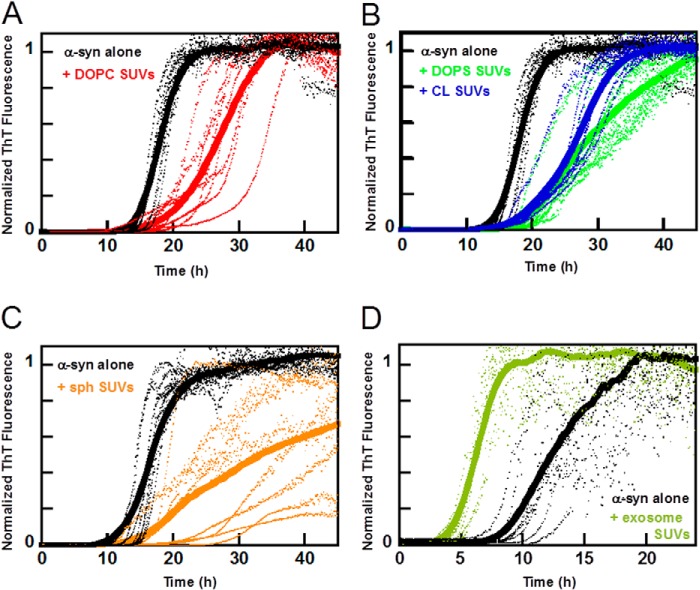
**Aggregation kinetics of α-syn in the presence of SUVs.** Aggregation kinetics for α-syn was measured by ThT fluorescence in the presence of SUVs in 10 mm MES, pH 5.5, with 140 mm NaCl. The averages of eight replicate traces are shown in *bold* with individual traces *dashed below. A,* aggregation of 30 μm α-syn (*black*) with 0.2 mm DOPC SUVs (*red*). *B,* aggregation of 30 μm α-syn (*black*) with 0.2 mm 30% DOPS SUVs (*green*) or 0.2 mm 6% cardiolipin SUVs (*blue*). *C,* aggregation of 30 μm α-syn (*black*) with 0.2 mm 15% sphingosine SUVs (*orange*). All mixtures are based on DOPC with addition of the indicated lipid. In all instances, the aggregation is retarded or shows no significant change in lag time when the lipid vesicles are added. *D,* aggregation of 30 μm α-syn alone (*black*) with extracted exosome lipid SUVs (*green*). Addition of SUVs made from extracted exosome lipids was the only model system studied that catalyzed the aggregation kinetics of α-syn.

The aggregation kinetics of α-syn was studied in the absence and presence of SUVs prepared from defined lipid mixtures (Fig. 6, *A–C*) or from lipid extracts of exosomes (Fig. 6*D*). α-syn aggregation is accelerated by the exosome lipid SUVs in a similar manner as observed with intact exosomes. This supports the notion that exosome lipid constituents play a role in the observed acceleration. However, most of the model lipid SUVs of similar size that we investigated did not accelerate α-syn aggregation, irrespective of lipid charge. Fig. 6, *A–C,* shows representative aggregation kinetics data for α-syn in the presence of SUVs composed of pure DOPC (uncharged, zwitterionic), DOPC with 6 mol % CL (net charge −2) (53), DOPC with 30 mol % DOPS (net charge −1), or DOPC with 15 mol % sphingosine (net charge +1). α-syn aggregation is not accelerated by any of these lipid systems, and in some cases we instead observe a minor retardation (Fig. 6, *A–C*). Thus, charge alone does not explain the acceleration of α-syn aggregation caused by the exosomes.

##### Effect of Lipid Concentration on α-Synuclein Aggregation Kinetics

**FIGURE 7.**
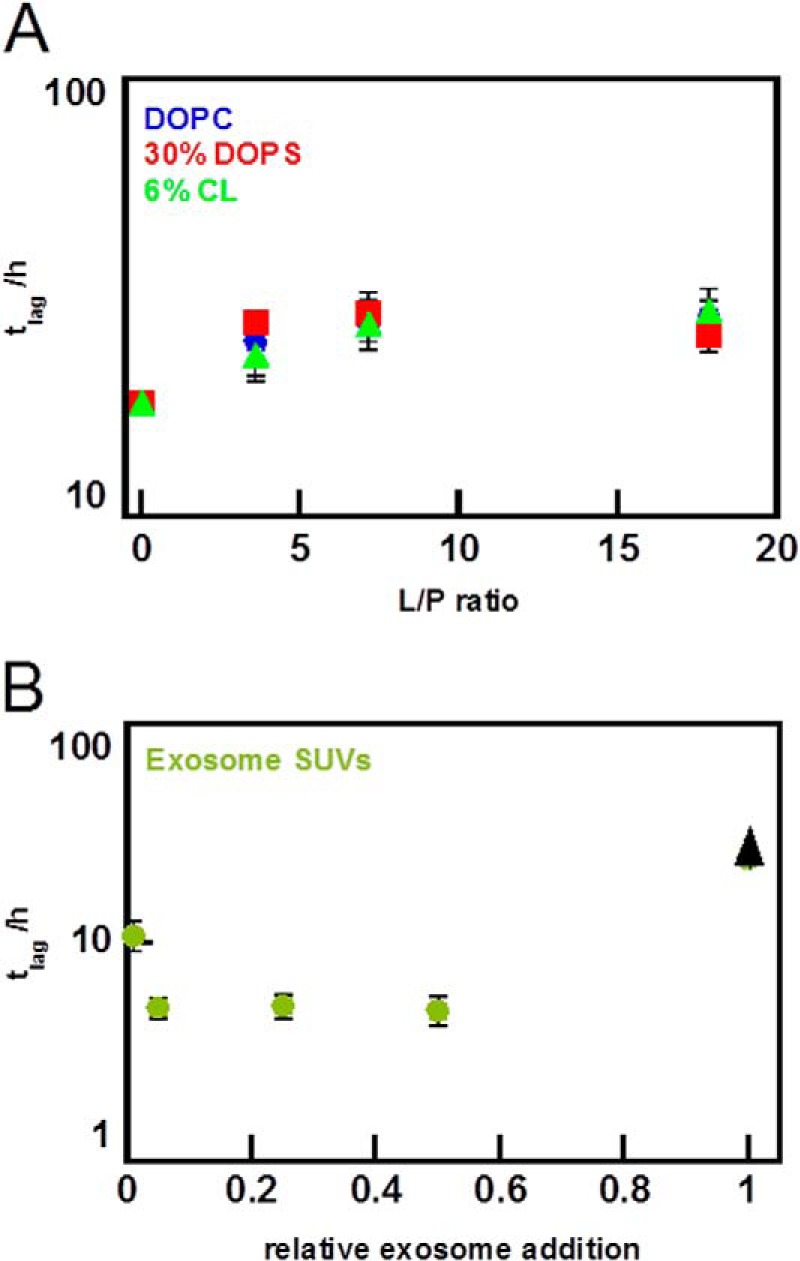
**Effect of lipid to protein ratio on aggregation lag time.** 30 μm α-syn was incubated in the presence of different concentrations of SUVs in 10 mm MES, pH 5.5, with 140 mm NaCl. The lag time is defined as the time when 10% of the maximum intensity is reached, and the *error bars* represent the standard deviation from eight replicates. All mixtures are based on DOPC with addition of the indicated lipid. *A,* lag time dependence for varying lipid to protein ratio for pure DOPC SUVs (*blue*), 30% DOPS SUVs (*red*), and 6% cardiolipin SUVs (*green*). Data collected at 100 rpm. *B,* lag time dependence for varying lipid to protein ratio for SUVs made from exosome lipids reported in relative lipid ratio. Addition of exosome SUVs decrease lag times until a threshold is reached and where the aggregation does not start within the experimental time frame (*black arrowhead*). Data were collected at 100 rpm.

Using a standard phosphorous assay (54), we estimated the lipid content in the exosome samples to 0.6 mm total lipids for exosome samples with total protein concentration (based on *A*_280_) of 1 mg/ml. The exosome concentration used in the aggregation assays then corresponds to ∼0.15 mm total lipid. Because this estimate cannot be considered to be very precise, we performed aggregation experiments with several concentrations of model vesicles (Fig. 7, *A* and *B*). This was done to investigate the role of available amounts of lipids or vesicle surface in the sample. We find that the lipid/protein ratio governs the effect on aggregation. At low lipid/protein ratios, the lag time is not significantly altered in the presence of DOPC, DOPS, or CL SUVs, although there is retardation in all lipid/protein ratios investigated with the stronger effect at higher lipid concentrations (Fig. 7*A*). In contrast, SUVs made of extracted exosome lipids accelerate aggregation except at the highest concentration where we did not observe any aggregation (Fig. 7*B*). Thus, under the present experimental conditions, none of the common membrane lipids DOPC, DOPS, or CL vesicles cause any acceleration at any concentration investigated, suggesting that other lipid species in the vesicles prepared from exosome-derived lipids are involved.

##### α-Synuclein Aggregation Kinetics in the Presence of Ganglioside Membranes

**FIGURE 8.**
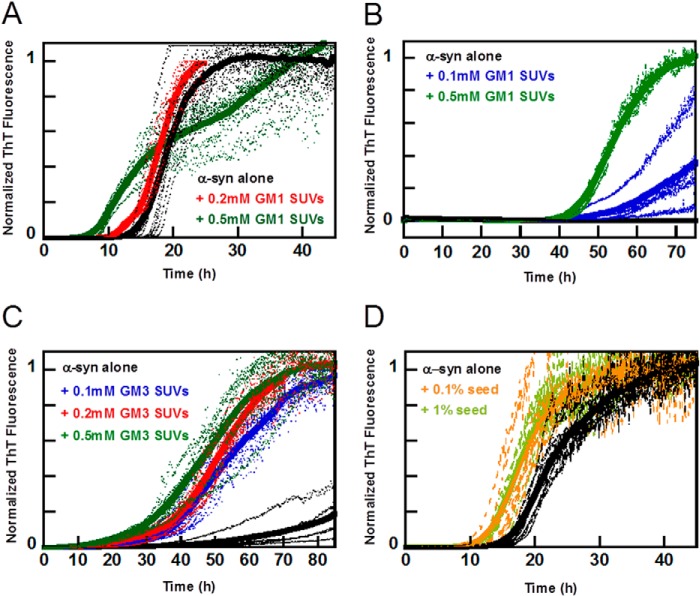
**Aggregation kinetics of α-syn in the presence of ganglioside SUVs or α-syn seeds.** Aggregation kinetics for α-syn was measured by ThT fluorescence in the presence of SUVs or α-syn seeds in 10 mm MES, pH 5.5, with 140 mm NaCl. The averages of eight replicate traces are shown in *bold* with individual traces *dashed below*. All mixtures are based on DOPC with addition of the indicated lipid. *A,* aggregation of α-syn (*black*) with 0.2 mm 10% GM1 SUVs (*red*) or 0.5 mm 10% GM1 (*green*) at 100 rpm. The biphasic behavior observed for 0.5 mm 10% GM1 SUVs is not reproduced in every experiment and may be due to the after-reaction of the formed fibrils (bundling and sedimentation, etc.) that perturbs the ThT fluorescence. *B,* aggregation of α-syn (*black*) with 0.1 mm 10% GM1 SUVs (*blue*) or 0.5 mm 10% GM1 (*green*) under quiescent conditions and in 96-well nonbinding pegylated surface plates. *C,* aggregation of α-syn (*black*) with 0.1 mm (*blue*), 0.2 mm (*red*), or 0.5 mm 10% GM3 SUVs (*green*) at quiescent conditions. *D,* aggregation of α-syn (*black*) with 0.1% (*orange*) or 1% seeds (*light green*) at 100 rpm.

The lipid analysis of isolated exosomes identified ganglioside classes GM2 and GM3. We therefore studied α-syn aggregation kinetics in the presence of model vesicles that include gangliosides. GM2 is not commercially available and could therefore not readily be investigated; however, both GM3 and GM2 are truncations of GM1, and as the three species have similar structures, we therefore studied aggregation kinetics in the presence of GM1 and GM3. Vesicles composed of DOPC with 10 mol % of GM3 or GM1 were prepared, and the effect of these vesicles on α-syn aggregation kinetics was investigated (Fig. 8, *A–C*). In sharp contrast to all other model lipid systems studied here (Figs. 6, *A–C,* and 7), the addition of these vesicles to the monomeric recombinant α-syn was found to accelerate the aggregation process in a concentration-dependent manner. All samples containing vesicles at 0.5 mm total lipid concentration exhibited faster aggregation than the samples containing vesicles at 0.2 mm total lipid concentration, which in turn aggregate faster than samples containing α-syn alone (Fig. 8, *A–C*). Clearly, vesicles composed of GM1 promote aggregation of α-syn also in nonbinding pegylated plates (Fig. 8*B*), where α-syn alone does not aggregate during the time frame acquired.

##### Aggregation in the Presence of Pre-formed Seeds

The addition of seeds composed of pre-formed amyloid fibrils is one efficient way to accelerate aggregation, due to secondary nucleation being favored at mildly acidic pH (35). Therefore, we compared the acceleration of amyloid formation observed with exosomes with that obtained with controlled amounts of seeds. Consequently, we studied the aggregation kinetics for samples that contain (at time 0) 30 μm α-syn monomer complemented with 0, 0.03, or 0.3 μm preformed α-syn fibrils (counted as monomer equivalents; Fig. 8*D*). At these low amounts of seeds (0.1 and 1%), elongation would be below the noise level of the ThT experiment. These small amounts of seeds are found to shorten the lag phase in a reproducible manner indicative of secondary nucleation of monomers on the fibril surface (44). In the presence of 1% seeds, the half-time has decreased by around 40% relative to the unseeded case. This reduction is slightly smaller than the reduction we observed for the samples containing exosomes.

**FIGURE 9.**
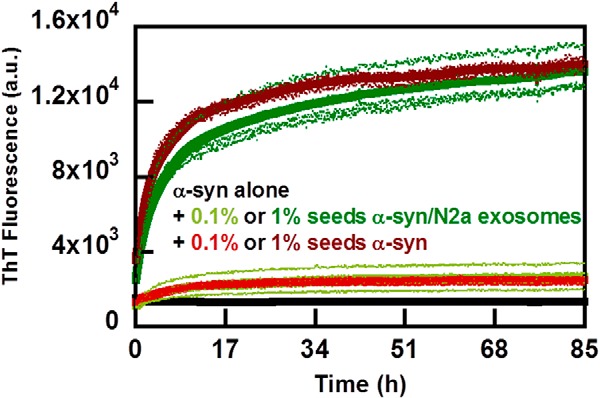
**Aggregation kinetics with seeds formed in the presence or absence of exosomes.** Aggregation of α-syn (*black*) was measured by ThT fluorescence in 10 mm MES, pH 5.5, with 140 mm NaCl in the presence of 0.1 or 1% seeds formed either with (*shades* of *green*) or without (*shades* of *red*) exosomes. The averages of four replicate traces are shown in *bold* with individual traces *dashed below*. Data were collected under quiescent conditions and in nonbinding pegylated surface plates.

We also studied whether seeds formed in the presence of exosomes have the same seeding efficiency as those formed in the absence of exosomes (Fig. 9). No discernable difference between the two seeds was observed.

##### Morphology of α-Synuclein Aggregates in the Presence of Exosomes and Vesicles

**FIGURE 10.**
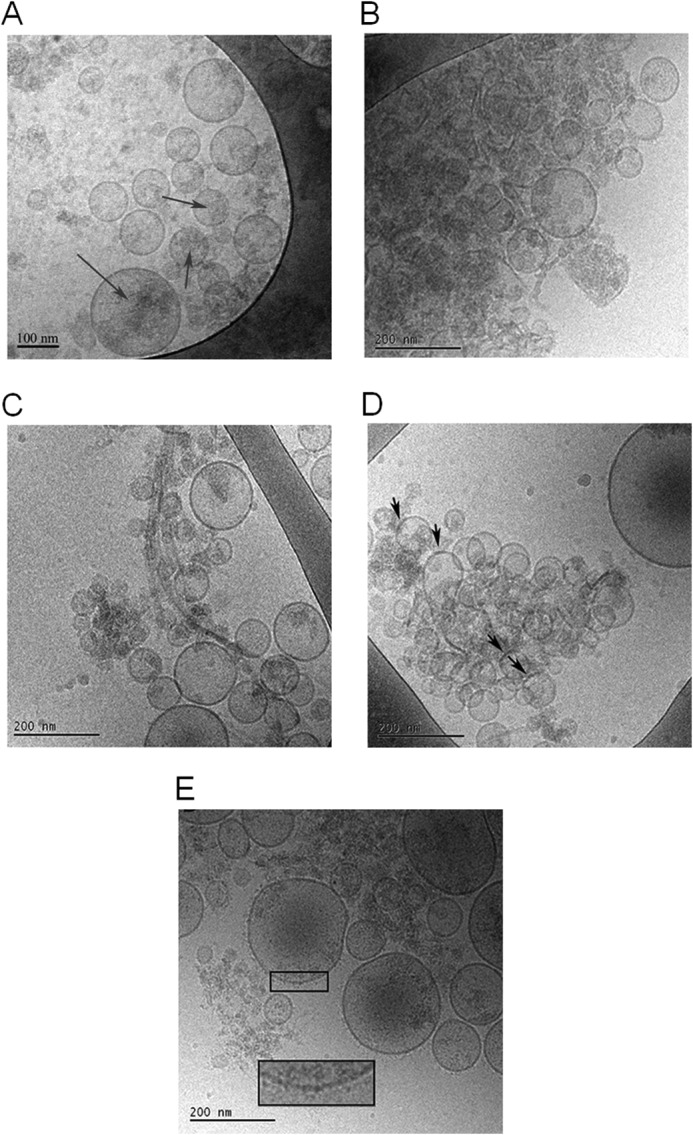
**cryo-TEM analysis of fibrils.** 5 μl of α-syn fibrils formed in 10 mm MES, pH 5.5, with 140 mm NaCl was added to the glow-discharged grid for cryo-TEM imaging. *A,* exosomes isolated from N2a cells overexpressing wild-type α-syn reveal spherical unilamellar vesicles with the presence of *darker gray areas* inside and on top of the vesicles (exemplified by *arrow*). *B,* when recombinant α-syn is mixed with exosomes from cells overexpressing α-syn, an immediate increase in “*gray shadows*” appears in the same area of the grid as where exosomes are found. *C,* after 18 h of co-incubation of recombinant α-syn with exosomes from cells overexpressing wild-type α-syn fibrils associated with the exosomes are present. *D,* incubation of recombinant α-syn with the exosomes leads to formation of patches in the exosome membrane that are darker than the surrounding membrane (marked by *arrows*). *E,* incubation of recombinant α-syn with exosomes overexpressing α-syn at neutral pH 7.5. Here a surface decoration of the exosome membrane is observed (*inset* shows magnification).

The cryo-TEM images of exosomes from cells overexpressing α-syn show small dark patches in the membrane and a small number of gray features in or around the exosomes when imaged directly after isolation (Fig. 10*A*). When recombinant α-syn is mixed with exosomes, there is an immediate increase in the number of gray patches around exosomes in the cryo-TEM images (Fig. 10*B*). After incubating the exosomes with recombinant α-syn for 18 h, fibrils are also observed in the sample (Fig. 10*C*). There is no striking difference in morphology between fibrils formed in the absence and presence of exosomes (Figs. 2*B* and 10*C*). Interestingly, the fibrils are observed in the same areas as the exosomes (Fig. 10*C*). Similar behavior is seen when α-syn is incubated with SUVs that contain anionic lipids (6 mol % CL) (data not shown). In the latter case, the α-syn fibrils entwine the SUVs and twist along the membrane surface. Incubating recombinant α-syn with exosomes leads to more prominent patches in the exosome membrane that are darker than the surrounding membrane (Fig. 10*D*). These patches may contain protein, protein aggregates, or some other components. Interestingly, when recombinant α-syn is incubated with exosomes at neutral pH 7.5 instead of pH 5.5, we observe a “surface decoration” of the exosome membrane (Fig. 10*E*).

## DISCUSSION

### 

#### 

##### Exosomes Accelerate Aggregation

Exosomes have been suggested to play a role in the transfer of α-syn between neurons, contributing to the spread of PD pathology between brain regions (27, 30–33). The results of our study show that aggregation of exogenous α-syn is accelerated by exosomes irrespective of whether they are derived from control cells or cells overexpressing α-syn (Fig. 3). Our results lead us to further suggest that the lipid composition of the exosomes is crucial for the aggregation process.

##### Lipid or Protein Component?

The acceleration of α-syn aggregation (Fig. 3*A*) might be caused by the exosome lipid or protein components or by some additional factor in the exosome samples. To distinguish between these possibilities, we studied the aggregation kinetics starting from monomeric α-syn samples supplemented with SUVs prepared from lipids extracted from the exosomes (Fig. 6*D*). Both N2a cell-derived exosomes and SUVs made of exosome lipids were found to accelerate α-syn aggregation. This implies that exosome lipids are sufficient for acceleration of α-syn aggregation and that the protein components of exosomes are not necessary for this effect to arise.

The observed kinetics are sensitive to the ratio of lipid to protein (Fig. 7). In our experiments, we had a molar excess of lipids to protein ranging from 4 to 18 lipids per protein molecule or 2 to 9 lipids per protein counting the outer leaflet only (most vesicles are unilamellar, Fig. 9) (55). An area of 72 Å^2^ per DOPC headgroup (56) gives 100–700 Å^2^ membrane surface area per protein molecule, which is less than the cross-section area of around 900 Å^2^ for a globular protein of 14 kDa and is significantly less than the area of unfolded α-syn monomer (57, 58) or α-syn with partial helical structure as in the proposed binding model (59). Thus, all protein may not adsorb in a single layer. This is in agreement with the findings of surface catalysis by nanoparticles when the protein is in excess over available surface area (60).

##### Membrane Charge and Lipid Composition

SUVs of similar size as the exosomes were prepared from pure lipids with variation of charge and lipid headgroup in a systematic manner. The lipids PC, PS, CL, and sphingosine were chosen for their biological relevance. PC is the most abundant lipid component of biological membranes, and the negatively charged CL and PS are relevant in PD and found in mitochondrial and plasma membranes. The amount of α-syn associated with mitochondria is increased in the substantia nigra of PD patients, and mitochondrial dysfunction is linked to PD pathogenesis (61). In addition to this, locally reduced pH occurs in mitochondria as a result of oxidative or metabolic stress (63), conditions relevant for PD. An acidic environment is also present in the endosomal-lysosomal pathway (62), which, as mentioned earlier, is heavily implicated in PD. Exosomes have been shown to be taken up by endocytosis (64) and as such are exposed to the acidic environments utilized in this study. Model vesicles containing negatively charged lipids were prepared to have similar charge density and ζ potential as the exosomes. We also tested model membranes with cationic lipids, although α-syn is known to preferentially interact with anionic lipids (65–67). Positively charged sphingosine is a sphingolipid precursor of cell signaling molecules such as ceramides and sphingosine phosphate (68). None of the model vesicles tested induce acceleration of α-syn aggregation (Fig. 6, *A–C*), in contrast to exosomes and SUVs prepared from exosome lipids, and both significantly accelerated α-syn aggregation (Figs. 1 and 6*D*). Vesicle charge seems to be of minor importance to the aggregation kinetics at the investigated concentrations and solution conditions. Thus, the mere presence of a phospholipid membrane is not sufficient to accelerate α-syn aggregation, irrespective of membrane charge.

##### Ganglioside Lipids

The lipid analysis of exosome extracts identified ganglioside lipids. Interestingly, the acceleration of α-syn aggregation as observed in the presence of exosomes is reproduced by vesicle preparations composed of fluid lipid bilayers that contain ganglioside lipids GM1 or GM3. The observed acceleration of α-syn aggregation in the presence of exosomes might thus be attributed to the lipid components GM2 and GM3, which are both truncated versions of GM1. Accelerated aggregation by ganglioside membranes has been observed for amyloid β (Aβ) from Alzheimer disease (AD) and islet amyloid polypeptide from diabetes type II (69–71). The opposite effect has been reported for α-syn, pH 7.5, in the presence of lipid model membranes containing ganglioside (57). This may be due to the difference in pH, lipid phase behavior (fluidity and domain formation) or other variations of the experimental setup. For example, the protein-to-lipid ratio, protein-to-surface area ratio, convection, and differences in surface material of the measuring cells, have all been shown to strongly affect α-syn surface nucleation (60).

##### Molecular Mechanism

Our results clearly show that the presence of exosomes affects the aggregation process and that the lipid composition is critical for the magnitude and nature of the effect. The underlying molecular mechanisms seem to rely on protein interaction with the lipid membrane. Still, many details remain elusive, and several scenarios might explain our observations. (i) Accumulation of protein at the bilayer interface may lead to surface catalysis and accelerated aggregation (60, 72). (ii) The adsorbed protein may bury hydrophobic segments in the apolar region of the bilayer, influencing the interactions with other protein molecules, and this may lead to catalysis or retardation of aggregation. (iii) Protein-lipid co-aggregation is another possible scenario that can lead to acceleration and retardation of the aggregation process. (iv) Protein adsorption to negatively charged ganglioside-containing membranes with large oligosaccharide headgroups might occur in a different manner compared with lipid bilayers with PS or CL where α-syn penetrates into interfacial headgroup region (45, 73). Lower acyl chain exposure may reduce the degree of protein penetration into the hydrophobic region of the membrane leading to conditions where surface catalysis is enhanced on ganglioside membranes.

##### Secondary Nucleation

In the seeding experiment, we observe a shortening of the lag phase, which indicates that the aggregation process is amplified by secondary nucleation, presumably of monomers on the fibril surface. This provides an autocatalytic feedback loop as recently found for Aβ (52, 74, 75). The α-syn aggregation process is highly pH-sensitive. At neutral pH under quiescent conditions, α-syn aggregation is extremely slow, and at mildly acidic pH (below pH 6), the rate of secondary nucleation increases dramatically, leading to much faster overall aggregation (35), presumably due to charge neutralization of monomer or fibril.

##### Exosomes and Other Amyloid Proteins

The interaction of another neurodegenerative disease-associated protein, Aβ, with gangliosides is well established (69, 76), and elevated ganglioside concentration is found in the brain and cerebrospinal fluid (CSF) of AD patients. Ganglioside-bound Aβ has been identified in CSF (77, 78) and is believed to be involved in seeding of amyloid fibrils in AD (70, 77, 79). Exosomes are suggested to play a role in AD pathogenesis, and ganglioside-containing fibrils are inferred to be toxic to cells (80, 81). Furthermore, in a mouse model of Sandhoff disease, a lysosomal disorder characterized by accumulation of GM2, it was observed that Aβ accumulated in the brains of these mice, whereasα-syn was accumulated in the substantia nigra (82). Lysosomal dysfunction is believed to play a role in PD (83, 84) and has been shown to enhance both α-syn and exosome secretion (32, 33, 41). In this study, we show that exosomes enhance the aggregation of α-syn, and this may be one factor inducing aggregation of extracellular α-syn. Given the role of these vesicles in transferring protein between cells, this then represents a viable pathway for spreading α-syn pathology in the PD brain. Indeed, this idea is supported by a recent study that shows monomeric Tau aggregated in endosomal components (85). Based on this, we may speculate that extracellular α-syn would interact with exosomes before being endocytosed; thereafter, it would encounter the acidic microenvironments of the endosomal pathway thus facilitating enhanced aggregation. Although we observed no enrichment of secreted α-syn on exosomes, the exosomes have a profound catalytic effect on α-syn aggregation kinetics, which we ascribe to the ganglioside lipid components. This, together with the autocatalytic multiplication of aggregates provided by the secondary nucleation at mildly acidic pH, provides important pieces of information in the complex puzzle of how α-syn interacts with biological membranes to promote neurological disease.

## Supplementary Material

Supplemental Data

## Footnotes

5 The abbreviations used are:

α-syn: α-synuclein
β-syn: β-synuclein
PC: phosphatidylcholine
PS: phosphatidylserine
PI: phosphatidylinositol
PE: phosphatidylethanolamine
CL: cardiolipin
DOPS: 1,2-dioleoyl-*sn*-glycero-3-phospho-l-serine sodium salt
DOPC: 1,2-dioleoyl-*sn*-glycero-3-phosphocholine
SUV: small unilamellar vesicle
PD: Parkinson disease
cryo-TEM: cryogenic transmission electron microscopy
DLS: dynamic light scattering
NTA: nanoparticle tracking analysis
AD: Alzheimer disease
amyloid β: amyloid β
ThT: thioflavin T
Hex: hexose.
