# Supplementary material for: Acceleration of α-Synuclein Aggregation by Exosomes
Source: J Biol Chem. 2014 Nov 25;290(5):2969–82. doi: 10.1074/jbc.M114.585703 (PMC4317028; doi:10.1074/jbc.M114.585703)
Supplement: Supplemental Data [file supp_290_5_2969__index.html]

Acceleration of α-Synuclein Aggregation by Exosomes — Exosomes Catalyze α-Synuclein Aggregation — Supplemental Data 

# Acceleration of α-Synuclein Aggregation by Exosomes

## Supplemental Data

**Files in this Data Supplement:**

- Supplemental Table
