# Supplementary material for: Acceleration of α-Synuclein Aggregation by Exosomes
Source: J Biol Chem. 2014 Nov 25;290(5):2969–82. doi: 10.1074/jbc.M114.585703 (PMC4317028; doi:10.1074/jbc.M114.585703)
Supplement: Supplemental Data [file supp_M114.585703_Grey_Table_SI1.docx]

Table SI1. Phospholipid characterization of exosomes. Phospholipid classes detected are phosphatidylethanolamine (PE), phosphatidylserine (PS), phosphatidylinositol (PI), phosphatidylcholine (PC) and the gangliosides GM2 and GM3. Fatty acid composition determined from MS^2^ in which the most intense FAs are shown in bold font. The relative intensity is calculated within each lipid class, where 100% represents the most abundant species found and ppm denotes the deviation of the experimental value from the theoretical.

| **Headgroup** | **Fatty acid** | **Lipid m/z** | **ppm** | **Relative abundance (%)** |
| --- | --- | --- | --- | --- |
| PE | **18:2-16:1** | 712,4929 | 0,9 | 3 |
|  | 18:3-16:0 |  |  |  |
|  | 14:0-20:3 |  |  |  |
| PE | **18:1-16:1** | 714,5091 | 1,6 | 29 |
|  | 18:2-16:0 |  |  |  |
|  | 14:0-20:2 |  |  |  |
| PE | **18:1-16:0** | 716,5252 | 2,3 | 35 |
|  | **18:0-16:1** |  |  |  |
|  | 14:0-20:1 |  |  |  |
| PE | **16:1-20:4** | 736,4938 | 2,1 | 3 |
|  | **16:0-20:5** |  |  |  |
| PE | **16:0-20:4** | 738,5103 | 3,2 | 16 |
|  | **16:1-20:3** |  |  |  |
| PE | **16:0-20:3** | 740,5245 | 1,2 | 29 |
|  | 16:1-20:2 |  |  |  |
| PE | **18:1-18:1** | 742,5403 | 1,4 | 100 |
|  | 18:0-18:2 |  |  |  |
|  | 16:1-20:1 |  |  |  |
|  | 16:0-20:2 |  |  |  |
| PE | **18:1-18:0** | 744,556 | 1,5 | 42 |
|  | 16:0-20:1 |  |  |  |
| PE | **18:1-20:5** | 762,5128 | 6,4 | 6 |
|  | 18:2-20:4 |  |  |  |
|  | **16:0-22:6** |  |  |  |
|  | **16:1-22:5** |  |  |  |
| PE | **18:1-20:4** | 764,525 | 1,9 | 31 |
|  | 18:0-20:5 |  |  |  |
|  | 18:2-20:3 |  |  |  |
|  | 16:0-22:5 |  |  |  |
|  | 16:1-22:4 |  |  |  |
| PE | **18:0-20:4** | 766,5408 | 2,1 | 71 |
|  | **18:1-20:3** |  |  |  |
|  | 18:2-20:2 |  |  |  |
|  | 16:1-22:3 |  |  |  |
|  | 16:0-22:4 |  |  |  |
| PE | **18:0-20:3** | 768,5562 | 1,7 | 51 |
|  | 18:1-20:2 |  |  |  |
|  | 18:2-20:1 |  |  |  |
|  | 16:0-22:3 |  |  |  |
| PE | **18:1-20:1** | 770,5708 | 0,4 | 15 |
|  | **18:0-20:2** |  |  |  |
|  | 16:1-22:1 |  |  |  |
|  | 16:0-22:2 |  |  |  |
|  | 16:1-22:1 |  |  |  |
| PE | **18:0-20:1** | 772,5858 | -0,5 | 5 |
|  | **18:1-20:0** |  |  |  |
|  | 16:0-22:1 |  |  |  |
| PE | **18:0-22:6** | 790,5532 | 17,7 | 5 |
|  | **18:1-22:5** |  |  |  |
|  | 16:1-24:5 |  |  |  |
| PE | **18:0-22:5** | 792,5578 | 3,7 | 17 |
|  | 18:1-22:4 |  |  |  |
|  | 18:2-22:3 |  |  |  |
| PE | **18:1-22:3** | 794,5474 | -29,1 | 21 |
|  | 18:0-22:4 |  |  |  |
|  | 18:2-22:2 |  |  |  |
| PE | **18:0-22:3** | 796,5864 | 0,3 | 10 |
|  | **18:1-22:2** |  |  |  |
|  | 18:2-22:1 |  |  |  |
|  | 16:1-24:2 |  |  |  |
| PE | **18:1-22:1** | 798,601 | -1,0 | 7 |
|  | **18:0-22:2** |  |  |  |
|  | 16:1-24:1 |  |  |  |
|  | 16:0-24:2 |  |  |  |
|  |  |  |  |  |
| PS | **16:0-18:1** | 760,5142 | 1,0 | 32 |
|  | **16:1-18:0** |  |  |  |
| PS | **18:1-18:1** | 786,53 | 1,2 | 24 |
|  | 18:0-18:2 |  |  |  |
| PS | **18:1-18:0** | 788,5454 | 0,9 | 100 |
|  | 16:0-20:1 |  |  |  |
|  | 16:1-20:0 |  |  |  |
| PS | **18:1-20:4** | 808,5122 | -1,5 | **6** |
|  | 18:0-20:5 |  |  |  |
|  | 16:0-22:5 |  |  |  |
|  | 16:1-22:4 |  |  |  |
| PS | **18:0-20:4** | 810,5278 | -1,5 | 21 |
|  | 18:1-20:3 |  |  |  |
|  | 18:2-20:2 |  |  |  |
|  | 16:0-22:4 |  |  |  |
|  | 16:1-22:3 |  |  |  |
| PS | **18:0-20:3** | 812,5451 | 0,5 | 7 |
|  | 18:1-20:2 |  |  |  |
|  | 16:1-22:2 |  |  |  |
|  | 18:2-20:1 |  |  |  |
| PS | **18:0-20:2** | 814,5612 | 1,0 | 16 |
|  | **18:1-20:1** |  |  |  |
|  | 16:1-22:1 |  |  |  |
|  | 16:0-22:2 |  |  |  |
|  | 18:2-20:0 |  |  |  |
| PS | **18:0-20:1** | 816,5772 | 1,5 | 13 |
|  | 18:1-20:0 |  |  |  |
|  | 16:1-22:0 |  |  |  |
|  | 16:0-22:1 |  |  |  |
| PS | **18:0-22:6** | 834,5298 | 0,9 | 15 |
|  | 18:1-22:5 |  |  |  |
| PS | **18:0-22:5** | 836,5443 | -0,5 | 17 |
|  | 18:1-22:4 |  |  |  |
| PS | **18:0-22:4** | 838,5589 | -1,7 | 4 |
|  | 18:1-22:3 |  |  |  |
| PS | **18:0-22:3** | 840,5768 | 0,9 | 7 |
|  | 18:1-22:2 |  |  |  |
| PS | **18:0-22:2** | 842,5931 | 1,7 | 13 |
|  | 18:1-22:1 |  |  |  |
|  | 18:2-22:0 |  |  |  |
| PS | **18:1-22:0** | 844,6096 | 2,7171 | 67 |
|  | **16:1-24:0** |  |  |  |
|  | 18:0-22:1 |  |  |  |
| PS | **18:0-24:4** | 866,5931 | 1,7 | 4 |
|  | 20:3-22:1 |  |  |  |
| PS | **18:1-24:2** | 868,6063 | -1,2 | **1** |
| PS | **18:1-24:1** | 870,625 | 2,3 | 4 |
|  | 18:0-24:2 |  |  |  |
|  | 18:2-24:0 |  |  |  |
|  | 16:1-26:0 |  |  |  |
|  | 16:0-26:1 |  |  |  |
| PS | **18:1-24:0** | 872,6402 | 1,8 | 7 |
|  | 18:0-24:1 |  |  |  |
|  |  |  |  |  |
| PI | **18:1-18:2** | 859,5332 | -1,2 | 9 |
|  | **16:0-20:3** |  |  |  |
|  | **18:0-18:3** |  |  |  |
| PI | **18:1-18:1** | 861,5509 | 1,2 | 71 |
|  | **18:0-18:2** |  |  |  |
|  | **16:0-20:2** |  |  |  |
| PI | **18:0:18:1** | 863,5665 | 1,1581 | 100 |
|  | 16:0-20:1 |  |  |  |
|  | 16:1-20:0 |  |  |  |
| PI | **18:0-20:4** | 885,5516 | 1,9763 | 36 |
|  | 18:1-20:3 |  |  |  |
| PI | **18:0-20:3** | 887,5667 | 1,3521 | 93 |
|  | 18:1-20:2 |  |  |  |
| PI | **18:0-20:2** | 889,582 | 0,9556 | 57 |
|  | 18:1-20:1 |  |  |  |
| PI | **18:0-20:1** | 891,5922 | -5,159 | 3 |
| PI | **18:0-22:2** | 917,609 | -3,76 | 2 |
|  |  |  |  |  |
| PC | **16:0-16:2** | 788,5456 | 1,1 | 49,7568709 |
|  | **16:1-16:1** |  |  |  |
|  | 14:1-18:1 |  |  |  |
| PC | **16:0-16:1** | 790,5606 | 0,3 | 20,6754118 |
|  | 14:0-18:1 |  |  |  |
|  | **16:0-16:1** |  |  |  |
|  | 14:0-18:1 |  |  |  |
| PC | **16:0-16:0** | 792,5767 | 0,9 | 19,8564604 |
|  | 14:0-18:0 |  |  |  |
|  | **16:0-16:0** |  |  |  |
|  | 14:0-18:0 |  |  |  |
| PC | **16:0-18:3** | 814,5616 | 1,5 | 5,65483871 |
|  | **14:0-20:3** |  |  |  |
|  | 16:1-18:2 |  |  |  |
| PC | **16:0-18:2** | 816,5775 | 1,8 | 13,9885561 |
|  | **16:1-18:1** |  |  |  |
|  | 14:0-20:2 |  |  |  |
| PC | **16:0-18:1** | 818,5914 | -0,3 | 100 |
|  | 16:1-18:0 |  |  |  |
|  | 14:0-20:1 |  |  |  |
| PC | **16:1-20:3** | 840,5774 | 1,7 | 4,56274874 |
|  | **16:0-20:4** |  |  |  |
|  | 14:0-22:4 |  |  |  |
| PC | **16:0-20:3** | 842,5932 | 1,8 | 9,99182312 |
|  | 16:1-20:2 |  |  |  |
|  | 14:0-22:3 |  |  |  |
|  | 18:1-18:2 |  |  |  |
| PC | **18:1-18:1** | 844,6091 | 2,1 | 5,06115421 |
|  | 16:0-20:2 |  |  |  |
|  | 18:0-18:2 |  |  |  |
| PC | **18:1-18:0** | 846,6242 | 1,5 | 50,1281755 |
|  | 16:0-20:1 |  |  |  |
|  | 16:1-20:0 |  |  |  |
|  |  |  |  |  |
| GM3 | 16:0 | 1151,708 | 1,8423 | 52 |
| GM3 | 20:0 | 1207,7705 | 1,674 | 7 |
| GM3 | 22:0 | 1235,8016 | 1,4742 | 9 |
| GM3 | 24:1 | 1261,8172 | 1,4042 | 12 |
| GM3 | 24:0 | 1263,8325 | 1,125 | 16 |
| GM2 | 16:0 | 1354,788 | 2,0297 | 100 |
| GM2 | 18:0 | 1382,8184 | 1,3377 | 8 |
| GM2 | 20:0 | 1410,85 | 1,5238 | 12 |
| GM2 | 22:0 | 1438,8826 | 2,3976 | 21 |
| GM2 | 24:1 | 1464,8979 | 2,1161 | 31 |
| GM2 | 24:0 | 1466,9118 | 0,9202 | 34 |
